# Associations between antibiotic consumption intensity and extended-spectrum cephalosporin resistance in nursing homes: a retrospective ecological study

**DOI:** 10.1093/jacamr/dlaf088

**Published:** 2025-05-23

**Authors:** Emmanouil Glampedakis, Anne Niquille, Patricia Cuiña Iglesias, Alessandro Cassini, Catherine Plüss-Suard, Andreas Kronenberg, Marie Immaculée Nahimana Tessemo, Tom R Brewer, Thomas Rawson

**Affiliations:** School of Public Health, Faculty of Medicine, Imperial College London, London, UK; Public Health Department, Cantonal Infection Prevention and Control Unit, Cantonal Doctor Office, Canton of Vaud, Lausanne, Switzerland; Department of Ambulatory Care, Unisanté, Center for Primary Care and Public Health and University of Lausanne, Lausanne, Switzerland; Institute of Pharmaceutical Sciences of Western Switzerland, University of Geneva, University of Lausanne, Geneva, Switzerland; Public Health Department, Cantonal Infection Prevention and Control Unit, Cantonal Doctor Office, Canton of Vaud, Lausanne, Switzerland; Public Health Department, Cantonal Doctor Office, Canton of Vaud, Lausanne, Switzerland; Infection Prevention and Control Unit, Infectious Diseases Service, Lausanne University Hospital and University of Lausanne, Lausanne, Switzerland; Swiss Centre for Antibiotic Resistance (ANRESIS), Institute for Infectious Diseases, University of Bern, Bern, Switzerland; Swiss Centre for Antibiotic Resistance (ANRESIS), Institute for Infectious Diseases, University of Bern, Bern, Switzerland; Public Health Department, Cantonal Infection Prevention and Control Unit, Cantonal Doctor Office, Canton of Vaud, Lausanne, Switzerland; School of Public Health, Faculty of Medicine, Imperial College London, London, UK; MRC Centre for Global Infectious Disease Analysis, Jameel Institute, School of Public Health, Imperial College London, London, UK

## Abstract

**Background:**

Studies relating the usage of antibiotics with extended-spectrum cephalosporin resistance (ESC-R) rates from clinical isolates in nursing homes (NHs) are rare. We investigated associations between the intensity of NH-level antibiotic consumption (ABC) and the frequency of ESC-R expressing urinary *Escherichia coli*, *Klebsiella* spp. and *Proteus* spp. isolates from NH residents.

**Materials and methods:**

We used retrospective data on ABC and ESC-R counts aggregated by NH and year between 2017 and 2022 from NHs of canton Vaud in Switzerland. Negative binomial regression was used to relate ABC intensity, expressed as DDDs per 1000 resident days, with counts of ESC-R expressing bacteria.

**Results:**

Fifty-four NHs were included cumulatively accounting for 6601 urinary isolates, of which 5028 *E. coli*, 999 *Klebsiella* spp. and 574 *Proteus* spp. Among these, the 6-year ESC-R cumulative incidence was 10.3% (*E. coli* 12.6%, *Klebsiella* spp. 3.8% and *Proteus* spp. 1.2%). Median annual overall ABC varied between 31.3 and 44.2 DDDs per 1000 resident days. There was no association between overall ABC, most antibiotic categories and ESC-R cumulative incidence. The consumption of cephalosporins [adjusted incidence rate ratio (aIRR): 1.023, 95% CI: 1–1.047] and carbapenems (aIRR: 1.542, 95% CI: 1.018–2.336) was independently associated with increased incidence.

**Conclusion:**

No association was found between overall ABC and ESC-R rates. Cephalosporin consumption showed a modest association, while for carbapenems this could reflect therapeutic use. These findings highlight the need for enhanced surveillance and resident-level data to better understand antibiotic resistance drivers in this setting.

## Introduction

Antimicrobial resistance (AMR) has been recognized as a major global public health threat by the WHO.^1^ Nursing home (NH) residents are prone to colonization and infection by MDR bacteria. In fact, evidence indicates that bacterial isolates from NH residents might exhibit higher rates of resistance compared to those from the ambulatory care setting.^2,3^ Consequently, NH residence has been incorporated into predictive algorithms for colonization by MDR pathogens.^4,5^ Although AMR in NHs remains an understudied topic, with data being scarce due to limited testing, some studies have documented rising AMR trends in NHs.^6,7^

Similarly, in Switzerland, recent research from NHs highlighted increasing rates of colonization by bacteria of the Enterobacteriaceae family expressing the extended-spectrum cephalosporin resistance (ESC-R) phenotype,^8^ which can cause urinary tract and other infections, and are associated with increased morbidity and mortality.^9^ ESBL encoded in plasmids are the more frequent resistance mechanisms behind the ESC-R phenotype. ESC-R management is challenging due to resistance to several first-line antibiotics, including most penicillins and cephalosporins. For instance, severe ESC-R infections might necessitate the use of broad-spectrum antibiotics, such as carbapenems.^10^ These therapies require intravenous administration making them less accessible in NHs, leading to hospital transfers, prolonged stays and higher costs. Furthermore, ESC-R resistance can spread between bacteria and individuals, resulting in outbreaks within healthcare settings, including NHs,^11^ posing infection control challenges for NHs and their residents.

Inappropriate and excessive antibiotic usage is a well-established driver of AMR development,^12^ as evidenced by studies conducted in both ambulatory^13,14^ and acute-care settings.^15^ In NHs, antibiotic exposure is common and often inappropriate, with residents frequently receiving more antibiotics than needed.^16^ This makes NHs key targets for antimicrobial stewardship (ABS) programmes.^17^ Nonetheless, while numerous studies report on AMR or antibiotic usage in NHs, these elements are often examined separately,^18^ so that associations between them remain uncharacterized in this setting. Generally, the same applies to research on the effectiveness of ABS programmes in NHs measuring AMR and/or antibiotic usage trends but lacking direct links between them.^19–21^ Investigating these relationships could provide valuable insights into the impact of ABS programmes and inform the prioritization of ABS initiatives in NHs.

This study aimed to investigate the relationship between antibiotic usage and the risk of carriage of the ESC-R phenotype in residents’ urine samples. The analysis focused on three clinically significant pathogen groups—*Escherichia coli*, *Klebsiella* spp. and *Proteus* spp.—which are the most common causes of NH urinary tract infections. Furthermore, we used antibiotic usage at an institutional level—hereafter referred to as antibiotic consumption (ABC)—which is more readily measurable than resident-level use and constitutes an essential component of NH antimicrobial stewardship.^22^ The primary objective was to assess the associations between ESC-R frequency and ABC intensity in NHs. A secondary objective involved comparing ESC-R trends over time in relation to longitudinal changes in ABC.

## Materials and methods

### Setting

The study was performed using data from NHs of the canton Vaud in Switzerland, one of the biggest Swiss cantons hosting 123 NHs and ∼6500 residents (68% female, mean age 87.7 years), as of 2023. NHs in Vaud are private non-profit and private for-profit institutions.^23^ Facility types include geriatric (hosting residents with aged-related physical dependency), psychogeriatric (hosting residents with neurodegenerative-related psychiatric conditions) and mixed institutions.^23^ These are further classified according to their location as urban (inside towns or cities), intermediate (outskirts of towns or cities) or rural. Medical care is provided by either personal general practitioners (GP) of residents or institution-employed GPs. These are responsible for antibiotic prescriptions and have free access to NH-tailored infection prevention and control, and treatment guidelines from the cantonal infection prevention and control unit of Vaud (HPCi Vaud) (available at www.hpci.ch). As NHs resemble common households, strict adherence to standard precautions is recommended for MDR bacteria carriers, without isolation measures.

### Study design and temporal extent

We conducted a retrospective ecological study using secondary data that have been collected from NHs of canton Vaud for surveillance purposes between 2017 and 2022. All data were collected in aggregated forms on a NH-level, which was our level of analysis.

### Datasets

We used two datasets: (i) a dataset of urinary cultures from HPCi Vaud, containing laboratory results of positive urinary cultures from NH residents. Each NH collaborates with a single, designated microbiological laboratory for their analyses, which transmits results to HPCi Vaud annually. Participating laboratories follow the EUCAST guidelines^24^ for antimicrobial susceptibility testing. In this study ESC-R was defined as phenotypic resistance to at least one third- or fourth-generation cephalosporin. The dataset contains numbers of microorganisms isolated annually in each NH and, when appropriate, the number among them expressing the ESC-R phenotype, as reported by the laboratories. In the case of cultures positive for up to three isolates, each of them was counted separately in their corresponding microorganism, and when appropriate, ESC-R categories. Cultures positive for more than three isolates were stored as ‘mixed flora’ without consideration (counting) of the implicated microorganisms in specific categories. Furthermore, preanalytical practices and clinical motivation behind urine sampling remained unknown. (ii) A dataset of drug consumption in NHs collected through NH-affiliated supplying pharmacies’ invoice data by Unisanté as part of the evaluation of the quality of care mandated by the cantonal health authorities. Data were obtained only on anti-infectives through the code J01 (antibacterials for systemic use), of the WHO Anatomical Therapeutic Chemical classification.^25^ We analysed the following antibacterial categories or molecules: penicillins, including their combination with beta-lactamase inhibitors (corresponding to the J01C code), cephalosporins [calculated after subtraction of the J01DH from J01D code, considering no consumption of monobactams (J01DF) and other cephalosporins and penems of the code J01DI in NHs], carbapenems (J01DH), quinolones (J01M), trimethoprim-sulfamethoxazole (TMP/SMX) (J01EE01), nitrofurantoin (J01XE01) and fosfomycin (J01XX01), which are among the most commonly prescribed antibacterials in NHs. Total ABC and consumption for each of these antibiotic categories were stored as the cumulative number of DDDs per NH over 1 year. The dataset contained the cumulative number of resident days per NH annually enabling the calculation of ABC intensity as DDDs per 1000 resident days. Given that new residents may receive antibiotics before institutionalization that would be impossible to exclude from our annual data, only consumption from residents present in the NH at the start of each year was taken into consideration and resident days were adjusted accordingly. The resulting ABC intensity represents 60% to 65% of participating NH residents. The dataset also contained information about the facility type (geriatric, psychogeriatric or mixed) and its localization (urban, intermediate or rural).

### NH inclusion criteria

We considered only the NHs that provided on-site medical care for residents aged 65 years and older and that had at least one data entry in both the urinary culture and the ABC datasets each year between 2017 and 2022. A flowchart of NH inclusions and exclusions with corresponding urinary culture and isolate numbers is provided in Figure S1 (available as Supplementary data at *JAC-AMR* Online).

### Statistical analysis

For our descriptive analyses we used numbers, percentages, medians and interquartile ranges (IQR) as needed. For the comparisons of categorical variables, we used Fisher’s exact test or the Chi-square test as appropriate, while the Wilcoxon test was used to compare continuous variables.

To evaluate the association between ABC intensities (total and category-specific) and ESC-R frequency in positive urinary cultures, we used hierarchical generalized linear models. To account for the nesting of repeated measures of ABC and resistance within NHs we constructed two-level negative binomial regression models with NHs as random intercepts. We first fitted a null model with the annual numbers of ESC-R expressing bacteria of interest (*Escherichia coli*, *Klebsiella* spp. and *Proteus* spp.) as the dependent variable and the natural logarithm of the total annual counts of bacteria of interest as an offset term. Second, we fitted separate univariable models, taking the null model and adding ABC intensities expressed as DDDs per 1000 resident days, facility type and NH localization as explanatory variables. Third, a multivariable model containing the ABC categories from previous models with significant *P* values, localization and facility type was fitted. Each model was compared to the null using the *F*-test. All models were fitted using maximum likelihood estimation with Laplace approximation using the glmmTMB R package.^26^ The coefficients of the models where exponentiated to represent incidence rate ratios with their 95% CI. The variance partition coefficient (VPC) represents the proportion of unexplained variance as a result of between NH differences and was calculated as previously described.^27^

To analyse the trend of ABC and resistance we summarized the ABC trend within each NH as the beta-coefficient of ordinary least square linear regression models having ABC per 1000 resident days as their dependent variable and time as their independent variable. For the resistance trends, we extracted beta coefficients from negative binomial regression models with dependent variable the numbers of ESC-R expressing bacteria and as independent variable time, including the natural logarithm of the total counts of bacteria of interest as an offset term. We then compared the number of NHs with concordant trends (same sign beta coefficients) among NHs with notable (*P* < 0.1) and non-significant ABC trends (*P* ≥ 0.1).

Results were statistically significant at *P* ≤ 0.05. Analyses were performed using R Statistical Software (version 4.3.3; R Foundation for Statistical Computing, Vienna, Austria) on RStudio.^28^

### Ethical statement

As individual-level data were not used, consent was not necessary. All analyses were based on data collected in aggregate forms on a NH-level, hence, the study fell out of the Swiss Human Research Act (CER-VD Req-2024-00186). The study was approved by the Research Governance and Integrity team of Imperial College, London (ICREC reference number 7038341).

## Results

### NH characteristics

Ninety-seven NHs had available urinary culture and/or ABC data between 2017 to 2022. Of these, 54 (56%) had complete microbiological and ABC data over the 6-year study period. Among institutions with complete data, 30 (56%) had urban, 20 (37%) intermediate and 4 (7%) rural localization. Twenty-six (48%) NHs were geriatric, 7 (13%) psychogeriatric and 21 (39%) mixed facilities. Table 1 summarizes the numbers of hosted residents and the corresponding annual resident days considered in the analysis over the study period. The median number of residents per institution ranged between 55.5 and 58.5. Comparisons with incomplete data institutions can be found in Tables S1 and S2. Institutions with incomplete data were mainly geriatric or psychogeriatric type and were hosting significantly lower numbers of residents throughout the study period compared to complete data NHs.

**Table 1.** Annual number of residents and resident days per NH included in the analysis

Year	Number of residents per NH median (IQR) (*n* = 54)	Resident days per NH median (IQR) (*n* = 54)
2017	57.00 (36.50)	16 961 (13 691)
2018	57.50 (36.00)	17 916 (12 530)
2019	57.50 (43.00)	17 530 (14 116)
2020	57.50 (49.50)	16 934 (15 248)
2021	55.50 (44.20)	17 775 (13 339)
2022	58.50 (38.80)	17 920 (11 008)

### Microbiological results

**Figure 1.**
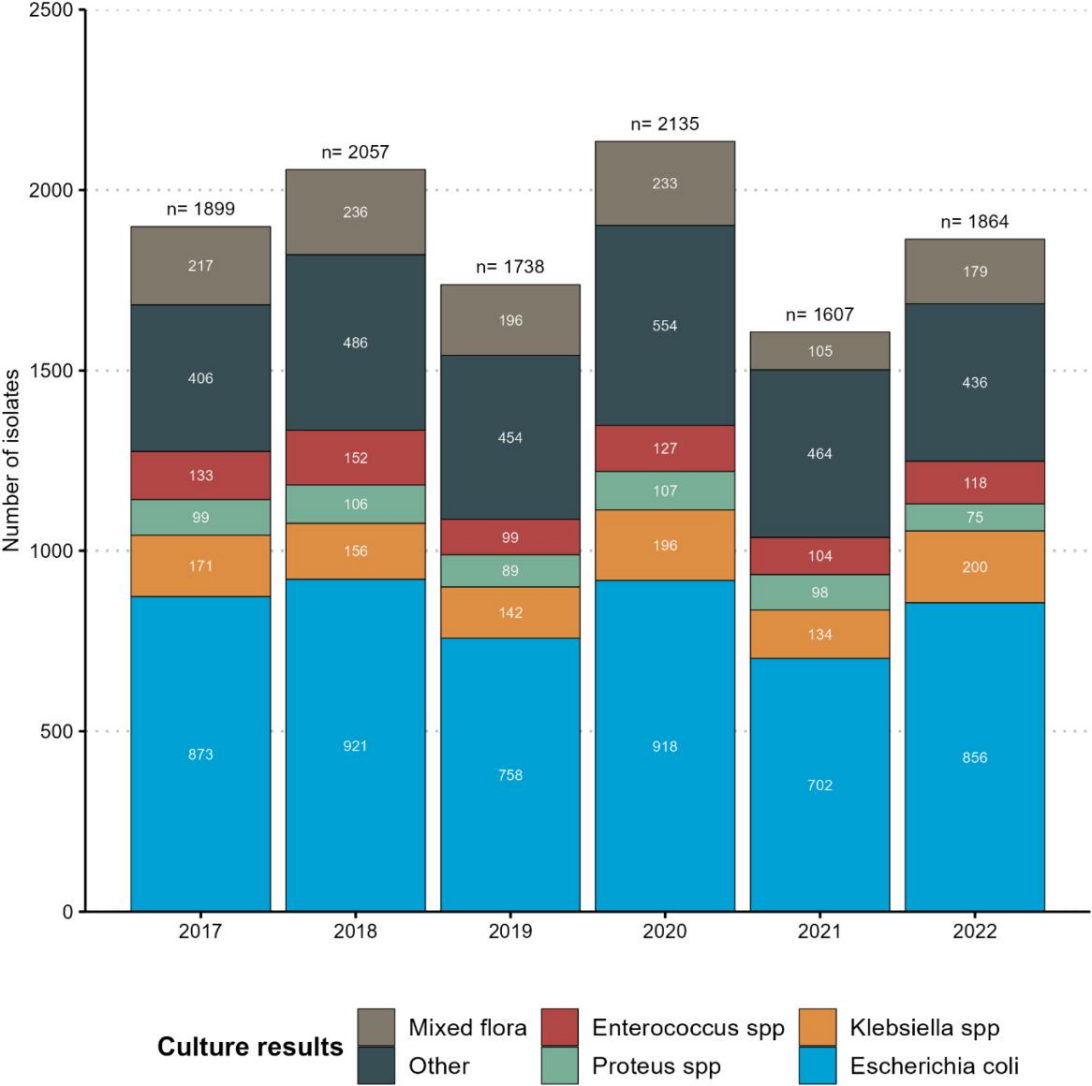
Bar plot summarizing urinary microbiological results over the 6-year period. Overall, there were 11 300 isolates from 9088 urinary cultures. Mixed flora (*n* = 1166): cultures positive for more than three microorganisms. Other microorganisms (*n* = 2800) included: *Streptococcus* spp. (*n* = 973), *Staphylococcus* spp. (*n* = 394), *Pseudomonas* spp. (*n* = 353), other Gram-positive cocci (*n* = 344), *Enterobacter* spp. (*n* = 251), other enterobacteria (*n* = 239), other Gram-positive bacteria (*n* = 152), other Gram-negative non-fermenters (*n* = 38), *Candida* spp. (*n* = 33), *Serratia* spp. (*n* = 17), anaerobic bacteria (*n* = 3), and non-identifiable (*n* = 3). *n*, Number; spp., Species.

In total, there were 9088 positive urinary cultures over the period 2017–2022 (mean of 0.56 positive cultures/resident) corresponding to 11 300 isolates (mean of 1.24 isolates/culture). Of all cultures, 66% were positive for one microorganism, 17% for two, 4% for three and 13% were classified as mixed flora (more than three microorganisms). Monobacterial positivity was 69% in 2017 and 2022 and varied between 63% and 68% in the intervening years. Details about resident urinary testing over time can be found in Table S3. Figure 1 illustrates detailed microbiological results grouped by year. Among the three bacteria of interest (*n* = 6601), *Escherichia coli* was the most frequently encountered (*n* =  5028, 76%), followed by *Klebsiella* spp. (*n* = 999, 15%) and *Proteus* spp. (*n* = 574, 9%).

### ESC-R frequency

**Figure 2.**
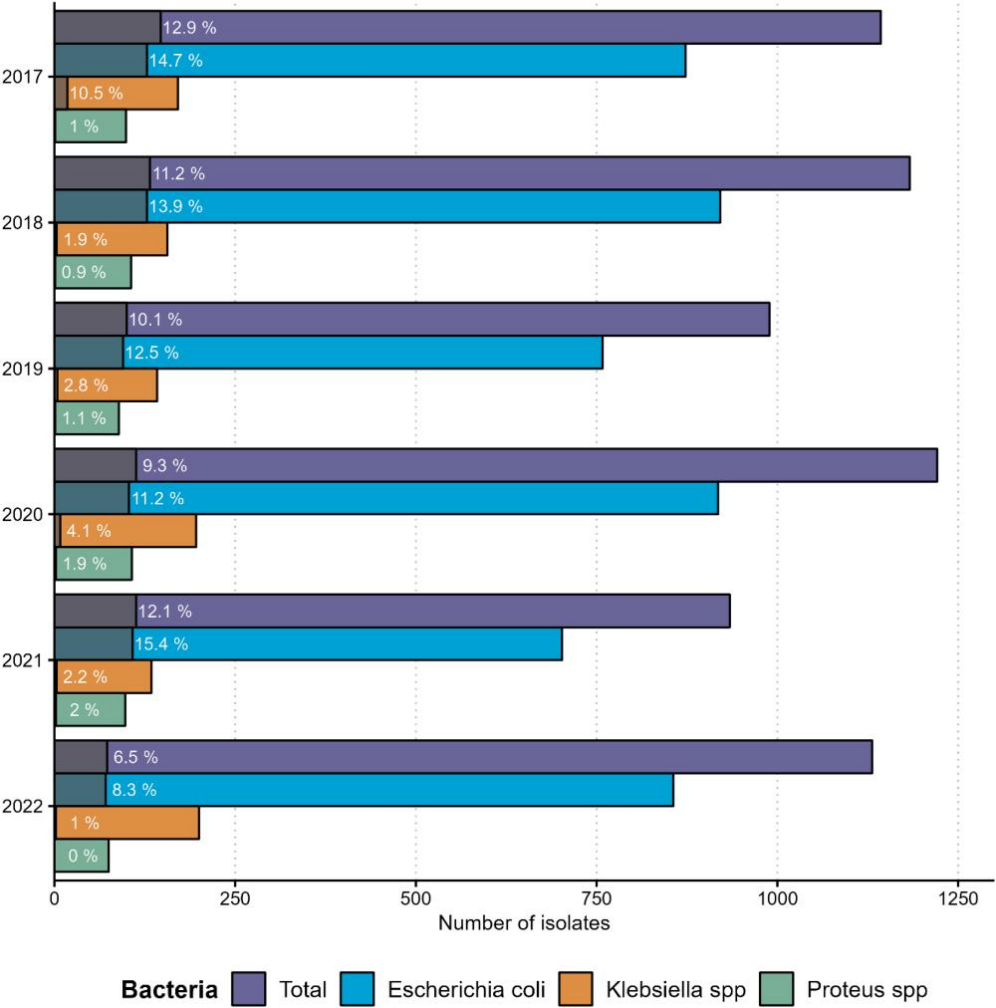
Frequency of bacteria of interest and corresponding counts and percentages of ESC-R expressing bacteria over the study period. Shaded colours inside the bars refer to counts and labels to percentages of corresponding ESC-R isolates. spp., Species.

The ESC-R phenotype was present in 678 of the bacteria of interest (10.3%). ESC-R frequency was higher among *Escherichia coli* isolates (*n* = 633/5028, 12.6%), compared to *Klebsiella* spp. (*n* = 38/999, 3.8%) and *Proteus* spp. (*n* = 7/574, 1.2%). Figure 2 shows the counts of ESC-R expressing among the three bacteria of interest and overall, along with their corresponding percentages. As demonstrated, ESC-R cumulative incidence varied from year to year but was lower in 2022 (6.5%) compared to 2017 and 2018. Figures S2 and S3 show microbiological results in NHs with incomplete data. Table 2 summarizes the evolution of distributions of ESC-R cumulative incidence over the 6-year period while similar results for incomplete data institutions can be found in Table S4.

**Table 2.** Distribution of ESC-R cumulative incidence among NHs over the study period

Year	Overall ESC-R percentage median (IQR) (*n* = 54)	*Escherichia coli* ESC-R percentage median (IQR) (*n* = 54)	*Klebsiella* spp. ESC-R percentage median (IQR) (*n* = 54)	*Proteus* spp. ESC-R percentage median (IQR) (*n* = 54)
2017	8.71% (14.50)	9.76% (18.80)	0.00% (0.00)	0.00% (0.00)
2018	5.44% (12.80)	6.90% (16.70)	0.00% (0.00)	0.00% (0.00)
2019	0.00% (10.30)	0.00% (12.30)	0.00% (0.00)	0.00% (0.00)
2020	2.86% (10.80)	1.43% (13.10)	0.00% (0.00)	0.00% (0.00)
2021	4.23% (9.88)	4.00% (16.70)	0.00% (0.00)	0.00% (0.00)
2022	3.34% (9.58)	4.55% (13.80)	0.00% (0.00)	0.00% (0.00)

### Antibiotic consumption

**Figure 3.**
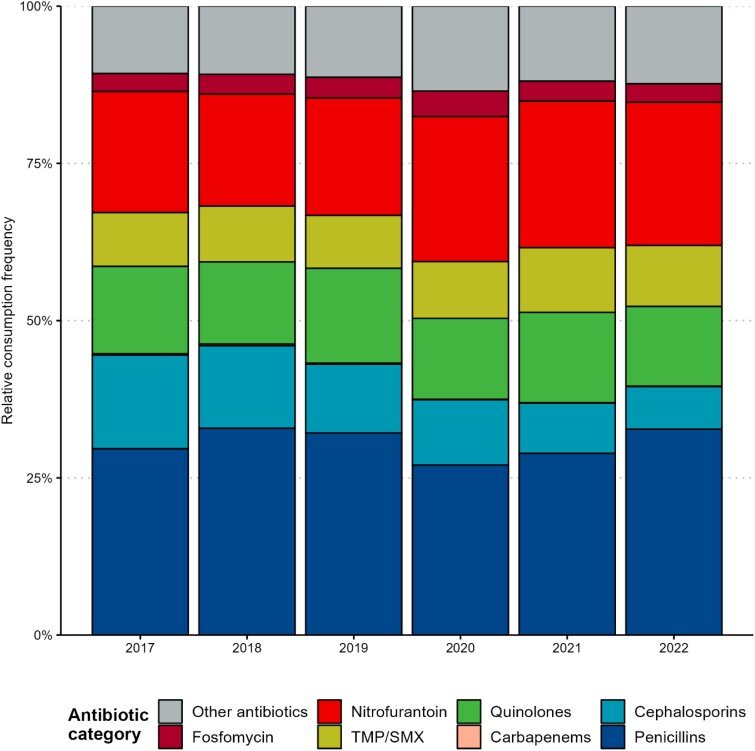
Relative consumption frequency of different antibiotic classes over the 6-year period. TMP/SMX, Trimethoprim/sulfamethoxazole.

Cumulatively, over the 6-year study period, penicillins were the most frequently consumed antibiotics (12.6 DDDs per 1000 resident days, 30.7% of the total resident-day adjusted ABC), followed by nitrofurantoin (8.67 DDDs per 1000 resident days, 21.1%), quinolones (5.40 DDDs per 1000 resident days, 13.2%), cephalosporins (4.19 DDDs per 1000 resident days, 10.2%), TMP/SMX (3.83 DDDs per 1000 resident days, 9.3%), fosfomycin (1.30 DDDs per 1000 resident days, 3.2%) and carbapenems (0.06 DDDs per 1000 resident days, 0.15%). Of all cephalosporin consumption, first-generation agents accounted for 0.1%, second-generation for 78.9%, third-generation for 20.9% and fourth-generation agents for 0.1%. There was a gradual decrease of ABC in the second half of the study period and total ABC fell from 41.4 DDDs in 2017 to 38.3 DDDs per 1000 resident days in 2022 (7.5% decrease). Figure 3 demonstrates the relative frequencies of consumed antibiotics while crude ABC can be found in Figure S4. Table 3 shows the distributions of DDDs consumed per 1000 resident days by antibiotic category and overall. Detailed characteristics of ABC in NHs with incomplete data can be found and Table S5 and Figures S5 and S6.

**Table 3.** Distributions of ABC intensity among NHs expressed as defined daily doses per 1000 resident days

Year	All antibiotics	Penicillins	Cephalosporins	Carbapenems	Quinolones	TMP/SMX	Nitrofurantoin	Fosfomycin
	DDDs per 1000 resident days, median (IQR) (*n* = 54)							
2017	39.60 (29.30)	11.40 (9.10)	2.82 (6.11)	0.00 (0.00)	4.31 (4.73)	2.49 (4.70)	4.79 (7.71)	0.74 (0.83)
2018	40.10 (17.80)	13.70 (7.41)	2.42 (7.33)	0.00 (0.00)	4.68 (3.89)	2.06 (4.10)	5.43 (8.47)	0.71 (1.26)
2019	44.20 (21.00)	13.00 (6.12)	4.12 (5.06)	0.00 (0.00)	4.70 (5.33)	2.91 (3.94)	6.25 (8.46)	0.81 (1.52)
2020	38.70 (19.20)	10.70 (6.95)	1.97 (2.82)	0.00 (0.00)	4.59 (3.61)	2.70 (5.02)	7.76 (8.70)	1.00 (1.45)
2021	38.00 (18.10)	9.43 (6.59)	1.69 (2.83)	0.00 (0.00)	4.57 (4.73)	3.01 (4.85)	7.85 (9.74)	0.60 (0.93)
2022	31.30 (26.70)	10.80 (6.74)	1.93 (2.46)	0.00 (0.00)	3.64 (4.04)	2.18 (3.93)	7.06 (10.80)	0.74 (0.99)

TMP/SMX, Trimethoprim/sulfamethoxazole.

### ESC-R incidence regression models

There was no significant association between the overall consumption of antibiotics and the cumulative incidence of urinary ESC-R bacteria. The results of the fixed effects of the univariable models are presented in Table 4. Univariable models revealed a significant positive association between the consumption intensities of cephalosporins and carbapenems and ESC-R frequency. Rural localization was associated with a 71.6% decrease of ESC-R incidence rates, compared to urban institutions. In the multivariable model presented in Table 5, the consumption intensities of cephalosporins (adjusted incidence rate ratio (aIRR): 1.023, 95% CI: 1–1.047) and carbapenems (aIRR: 1.542, 95% CI: 1.018–2.336) were independently associated with increased ESC-R incidence. On the contrary, rural localization was independently associated with decreased ESC-R incidence rates (aIRR: 0.299, 95% CI: 0.104–0.857). The multi-level analysis showed that most ESC-R variance could be attributed to within-NH characteristics, with median VPCs ranging between 0.119 and 0.170.

**Table 4.** Univariable models relating the intensity of ABC and other NH characteristics with the cumulative incidence of ESC-R in urines

Model	Intercept 95% CI	IRR 95% CI	VPC Median (Q1-Q3)	Median VPC percentage change^a^	*R* ^2^	*R* ^2^ percentage change^a^	*P* value^a^
**ABC models**							
Null model	0.079 (0.064–0.096)	—	0.165 (0.095–0.218)	—	0.077	—	—
Overall ABC	0.074 (0.051–0.107)	1.002 (0.993- 1.010)	0.156 (0.089–0.205)	−5.45%	0.073	−5.19%	0.702
Penicillins	0.085 (0.062–0.116)	0.993 (0.972–1.015)	0.170 (0.100–0.230)	+3.03%	0.081	+5.19%	0.546
Cephalosporins	0.070 (0.056–0.088)	1.024 (1.000–1.048)	0.164 (0.091–0.216)	−0.61%	0.082	+6.49%	**0**.**046**
Carbapenems	0.076 (0.062–0.092)	1.597 (1.042–2.447)	0.151 (0.086–0.205)	−8.48%	0.075	−2.60%	**0**.**026**
Quinolones	0.074 (0.057–0.096)	1.012 (0.977–1.048)	0.155 (0.089–0.204)	−6.06%	0.073	−5.19%	0.519
TMP/SMX	0.082 (0.065–0.104)	0.987 (0.953–1.023)	0.170 (0.100–0.230)	+3.03%	0.081	+5.19%	0.483
Nitrofurantoin	0.074 (0.058–0.094)	1.008 (0.990–1.026)	0.153 (0.087–0.202)	−7.27%	0.072	−6.49%	0.409
Fosfomycin	0.073 (0.058–0.091)	1.062 (0.972–1.160)	0.147 (0.083–0.196)	−10.91%	0.072	−6.49%	0.189
**Type of facility models (ref. = geriatric type)**							
Mixed	0.088 (0.068–0.114)	0.916 (0.621–1.350)	0.150 (0.080–0.190)	−9.09%	0.080	+3.90%	0.151
Psychogeriatric		0.545 (0.297–1.003)					
**Localization models (ref. = urban localization)**							
Intermediate	0.088 (0.069–0.110)	0.864 (0.586–1.274)	0.149 (0.078–0.198)	−9.70%	0.103	+33.77%	**0**.**040**
Rural		0.284 (0.098–0.824)					

Bold *P*-values indicate statistical significance. *R*^2^ refers to the proportion of variance explained by the fixed and random effects of the model, calculated as previously described.^29^

ref., reference category; TMP/SMX, trimethoprim/sulfamethoxazole.

^a^Compared to the null model.

**Table 5.** Multivariable model associating the selected predictors with the cumulative incidence of ESC-R in urines

Predictor	Intercept 95% CI	aIRR 95% CI	VPC Median (Q1-Q3)	Median VPC percentage change^a^	*R* ^2^	*R* ^2^ percentage change^a^	*P* value^a^
Cephalosporins	0.088 (0.067–0.116)	1.023 (1.000–1.047)	0.119 (0.056–0.158)	−27.88%	0.112	+45.45%	**0.005**
Carbapenems		1.542 (1.018–2.336)					
Mixed type		0.831 (0.567–1.217)					
Psychogeriatric type		0.571 (0.317–1.028)					
Intermediate localization		0.828 (0.565–1.213)					
Rural localization		0.299 (0.104–0.857)					

Bold *P*-values indicate statistical significance. Geriatric type and urban localization facilities have been taken as reference.

*R* ^2^ refers to the proportion of variance explained by the fixed and random effects of the model, calculated as previously described.^29^

^a^Compared to the null model of Table 4.

### Longitudinal antibiotic consumption and ESC-R analysis

The results of the longitudinal trend analysis are presented in Table 6. NHs with *P* values < 0.1 in longitudinal ABC intensity trends did not show more concordant trends in ESC-R evolution compared to institutions with *P* ≥ 0.1. Generally, the proportions of those showing concordant ABC/ESC-R-resistance trends were higher among NHs with notable (*P* < 0.1) cephalosporin, carbapenem and quinolone consumption changes over time, yet only the cephalosporin group approached statistical significance (*P* = 0.06).

**Table 6.** Comparisons between NHs with respect to their ABC longitudinal trends

Antibiotic class	Concordant^a^ ABC and ESC-R trends, *N* (%) NHs with non-significant^b^ ABC trends	Concordant^a^ ABC and ESC-R trends, *N* (%) NHs with notable^c^ ABC trends	*P* value^d^
All antibiotics	18/37 (49)	6/13 (46)	1.000
Penicillins	17/40 (42)	5/10 (50)	0.732
Cephalosporins	16/36 (44)	11/14 (79)	0.056
Carbapenems	11/16 (69)^e^	1/1 (100)^e^	1.000
Quinolones	18/42 (43)	5/8 (62)	0.444
TMP/SMX	19/43 (44)	2/7 (29)	0.684
Nitrofurantoin	17/37 (46)	5/13 (38)	0.751
Fosfomycin	23/37 (62)	8/13 (62)	1.000

Analysis on 54 NHs with complete data (50 units of analysis).

TMP/SMX, trimethoprim-sulfamethoxazole.

^a^NHs with similar sign beta coefficients in longitudinal trend analysis (increasing ABC and increasing ESC-R or decreasing ABC and decreasing ESC-R counts).

^b^ *P* ≥ 0.1 in the ABC longitudinal trend analysis.

^c^ *P* < 0.1 in the ABC longitudinal trend analysis.

^d^Fisher’s exact test.

^e^33 NHs excluded because of zero beta coefficients in their carbapenem longitudinal trend analysis.

## Discussion

We aimed to investigate associations between ABC and AMR in NHs using aggregated surveillance data from canton Vaud, Switzerland. We focused on three clinically important urinary tract bacteria and ESC-R phenotype, both of which carry important health and quality-of-life implications for NH residents. We found no association between overall ABC and ESC-R incidence, but positive associations were observed for cephalosporins and carbapenems. Longitudinal trend analysis did not reveal a clear impact of overall ABC on ESC-R frequency over time. However, for some antibiotic categories, proportions of NHs demonstrating aligned ABC-ESC-R trends were higher when the ABC longitudinal trend was more pronounced.

Previous studies have established links between ABC and AMR across various pathogens in both community^13,14^ and acute-care settings.^15^ However, extrapolating such findings to the NH context may be challenging because, while NH residents are generally at higher risk of AMR carriage than community patients,^2–5^ they may still be at lower risk compared to those receiving hospital care. We analysed ABC-AMR associations using aggregated, NH-level data that are routinely collected and encouraged within NH antibiotic stewardship initiatives.^22^ A MEDLINE search with the keywords (‘ESC-R’ OR ‘ESCR’ OR ‘ESBL’ OR ‘beta-lactamase’ OR ‘cephalosporin’) AND (‘Nursing home’ OR ‘long-term care’) highlighted the paucity of studies employing a similar methodology, making the current study one of the few examining ABC-ESC-R relationships through aggregated data. In parallel, studies using resident-level data have identified significant links between antibiotic exposure of any type,^30–33^ especially within the preceding 6 months, and ESC-R carriage. These discrepancies imply that resident-level analyses, by capturing more granular details, might be more sensitive in detecting associations, whereas with the ecological nature of our approach effects might have been diluted and potential relationships attenuated.

Our models revealed positive associations between the cephalosporin consumption, mainly represented by second-generation (78.9%) and third-generation agents (20.9%), and ESC-R counts in resident urinary cultures. These results align with previous reports identifying cephalosporins as an important risk factor for ESC-R carriage in the long-term care setting.^34,35^ They also support current recommendations to limit cephalosporin use in NHs to reduce cephalosporin resistance burden.^36^ Furthermore, our models demonstrated significant positive associations between carbapenem consumption and urine ESC-R incidence. Nonetheless, the wide confidence interval for carbapenems reflects the high degree of uncertainty related to the infrequent usage of these antibiotics. Since carbapenems are among the antibiotics used to treat ESC-R infections,^10^ we cannot affirm relationships between carbapenem exposure and ESC-R incidence, as higher consumption may merely reflect heightened therapeutic activity in response to existing ESC-R cases (reverse causality). Such a confounding scenario is less plausible for cephalosporins given their limited efficacy against ESC-R infections. Interestingly, and similar to other studies in long-term care^30,32^ we did not observed the association between the use of quinolones and ESC-R incidence that has been reported in other settings.^37^

The output from the multivariate model suggested that an average NH would experience a 2.3% decrease in ESC-R incidence following a one DDD per 1000 resident days reduction in cephalosporin consumption. For example, consider a NH with above-median cephalosporin use, consuming four DDDs per 1000 resident days and having a baseline cumulative ESC-R incidence of 15%. Reducing cephalosporin consumption by three DDDs per 1000 resident days (a 75% reduction) would result in only a modest decrease in ESC-R incidence, from 15% to ∼14%. These findings suggest a rather small effect size and complement the previously mentioned cautions on the use of NH-level consumption data to predict ESC-R frequencies or to inform ESC-R reduction policies. Although the effect size may limit direct policy implications, it underscores the need for more in-depth analyses of the impact of cephalosporins on ESC-R to better guide future ABS interventions in NHs.

The present study also revealed that NH characteristics other than institutional ABC may influence ESC-R rates in clinical samples. Although the exact underlying mechanisms remain to be elucidated, one plausible explanation is that different types of institutions care for residents with varying risk profiles. Indeed, previous research has reported differences in AMR carriage attributable to the type of long-term care facility.^38,39^ Here we observed significantly lower ESC-R rates in rural NHs compared with the urban ones. Healthcare associated-infections are less common in rural NHs,^40,41^ a finding that may be linked to reduced antibiotic exposure and, consequently, diminished selection pressure. In the same line, a recent report from Switzerland has demonstrated that residents in rural long-term care facilities tend to receive less antibiotics.^42^ Moreover, residents in rural facilities may have fewer contacts with hospital environments, thereby lowering the risk of colonization by resistant bacteria.^43^

The success of ABS programmes depends on sustained reductions in ABC that are followed by similar decreases in AMR rates. Our study proposes a method to compare AMR trends between NHs based on their ABC evolution over time. Although we did not find notable differences between institutions with significant and non-significant overall ABC trends, AMR-ABC trends tended to align more frequently when the ABC changes were pronounced, particularly for cephalosporins, carbapenems and quinolones. These observations are partly consistent with our model results and warrant further investigation in future research projects.

From an AMR surveillance perspective, previous research has documented high ESC-R rates in long-term care settings.^6,7^ In addition, a Swiss study involving NHs in canton Vaud reported increasing prevalence among residents between 2007 and 2017.^8^ In our analysis, we observed varying levels of annual ESC-R frequency, with an overall cumulative incidence of 10.3% over the 6-year period, remaining lower over time than previously reported values from Switzerland.^8^ Moreover, ESC-R rates in our study were lower at the end (2021–2022) compared to the beginning of the study period (2017–2018). This finding may be attributable to the SARS-CoV-2 pandemic and the related mitigation measures. A similar trend was reported in a French study, which observed decreasing cephalosporin resistance rates in NHs during the pandemic period.^44^ It is noteworthy that part of the heterogeneity of the literature-reported ESC-R frequencies may be explained by differences between studies reliant on clinically-motivated samples^8^ and those reporting colonization rates among asymptomatic residents.^6,45^ As urinary cultures are generally performed in response to a clinical suspicion of infection, the data used in this study may more accurately reflect the morbidity burden of urinary pathogens for NH residents.

Since 2011, ABS initiatives in canton Vaud, including the introduction of guidelines for infection treatment customized to the NH setting, and the implementation of physician-pharmacist-nurse quality circles^46^ to ensure their implementation, have contributed to a gradual decline in antibiotic use.^23^ Our findings indicate that overall ABC remained stable during the first half of the study period, while both crude and adjusted consumption gradually decreased thereafter. The reductions primarily concerned penicillins, quinolones and cephalosporins, while an increase in nitrofurantoin use was observed. Similar reductions in NH ABC have been documented in other regions following the SARS-CoV-2 pandemic.^47,48^ Continued monitoring will be necessary to determine whether these observed trends persist in the post-pandemic era.

Our study has several limitations: (i) it used aggregated, NH-level data, precluding the consideration of various clinical parameters known to influence resistant pathogen carriage. Notably, the ecological design made it impossible to ascertain whether resistance occurred in residents who received the antibiotics or the rest. (ii) Factors that could have influenced ESC-R carriage, and include resident demographics, comorbidities, functional status, prior hospitalization, pressure ulcers and urinary catheters,^31,32,49,50^ as well as infection control practices such as hand hygiene staff adherence, could not be accounted for in this study. It is noteworthy that the omission of these factors could explain the high proportion of ESC-R rate variability that remained unaccounted for by the models (relatively low *R*^2^). The multi-level analysis revealed that most of the unexplained variance in ESC-R rates is attributable to time-varying factors within NHs. Such unexplored variables could include the numbers of previously identified ESC-R carriers that could serve as reservoirs for ongoing dissemination and were impossible to incorporate in the present analysis. Future investigations incorporating these previously mentioned factors as covariates could more accurately elucidate the true extent of the net ABC influence on the risk of ESC-R carriage and are therefore strongly encouraged. (iii) As our study focused on NHs within a single geographical region, generalizability may be limited, and future research should consider multicentre data from multiple countries to enhance external validity. Furthermore, analyses presented in Supplementary material indicate that the results of the present study primarily stem from relatively large NHs. Further confirmation of these results in smaller institutions, as well as in other geographical and resource settings, is warranted. (iv) Our ABC data capture ∼60%–65% of participating NH residents and may not fully represent facilities with high resident turnover or those with a greater proportion of short-term stays. (v) Although still appropriate for the objectives of this study, our phenotypic ESC-R definition did not account for the underlying resistance mechanisms (i.e. ESBL or AmpC), which may limit comparability with studies that specifically address ESBL and AmpC producers using genotypic methods.

In conclusion, this study is among the few examining the ecological associations between NH-level ABC intensity and urinary ESC-R rates. While overall ABC did not significantly influence ESC-R frequency, cephalosporin consumption showed modest positive associations, and carbapenem usage was associated with increased ESC-R resistance, possibly reflecting therapeutic use rather than causal effects. Overall, the current work underscores the need for enhanced surveillance systems and more granular, resident-level data to predict ESC-R rates in this vulnerable population.

## Supplementary Material

dlaf088_Supplementary_Data

## Data Availability

Data (anonymized for NHs) and R code used to run the analyses might be obtained on reasonable request to the corresponding author.

## Supplementary data

Figures S1–S6 and Tables S1–S5 are available as Supplementary data at *JAC-AMR* Online.
