# Supplementary material for: Associations between antibiotic consumption intensity and extended-spectrum cephalosporin resistance in nursing homes: a retrospective ecological study
Source: JAC Antimicrob Resist. 2025 May 23;7(3):dlaf088. doi: 10.1093/jacamr/dlaf088 (PMC12099033; doi:10.1093/jacamr/dlaf088)
Supplement: dlaf088_Supplementary_Data [file dlaf088_supplementary_data.docx]

**SUPLEMENTARY MATERIAL**

**SUPPLEMENTARY FIGURES**

**Figure S1.** Flowchart of institution, urinary cultures, and urinary isolates

inclusions/exclusions

**Urinary isolates**

**Urinary cultures**

12 NHs provided grouped urinary results (by 2)

Antibiotic consumption database

Urinary culture database

19 NHs not present in urinary culture database

-12 geriatric

-4 psychogeriatric

-3 mixed type

15’527 positive cultures

16’062 positive cultures

16’290 positive cultures

**97 NHs / 91 UoA
U**

**54 NHs / 50 UoA**

**with complete data**

**54 NHs / 50 UoA**

**with complete data**

102 NHs in both databases

**Institutions**

10 LTCFs for adults <65 years old

117 LTCFs

127 LTCFs

121 LTCFs

143 LTCFs

22 LTCFs for adults <65 years old

19’813 isolates

19’492 isolates

15 LTCFs not present in antibiotic database:

-4 not in canton Vaud

-2 non-medicalized resting homes

-5 LTCFs for adults <65 years old

-4 geriatric type NHs

**11’300 isolates**

**6’601 isolates of interest**

- **5’028 *E. coli***
- **999 *Klebsiella* spp.**
- **574 *Proteus* spp.**

**9’088 positive cultures**

**11’300 isolates**

**9’088 positive cultures**

**14’926 isolates**

**12’144 positive cultures**

18’831 isolates

18’831 isolates

15’527 positive cultures

Grouping of antibiotic consumption in 12 NHs (by 2)

5 NHs / 5 UoA with no data between 2017-2022

102 NHs / 96 UoA

***Descriptive analyses***

43 NHs / 41 UoA

with incomplete urinary and/or antibiotic data

***Primary and secondary***

***objective analyses***

Figure S1: LTCF: long-term care facility, NH: nursing home, spp: Species, UoA: Units of analysis

**Figure S2.** Bar plot summarizing urinary microbiological results over the six-year period in the 43 NHs with incomplete data


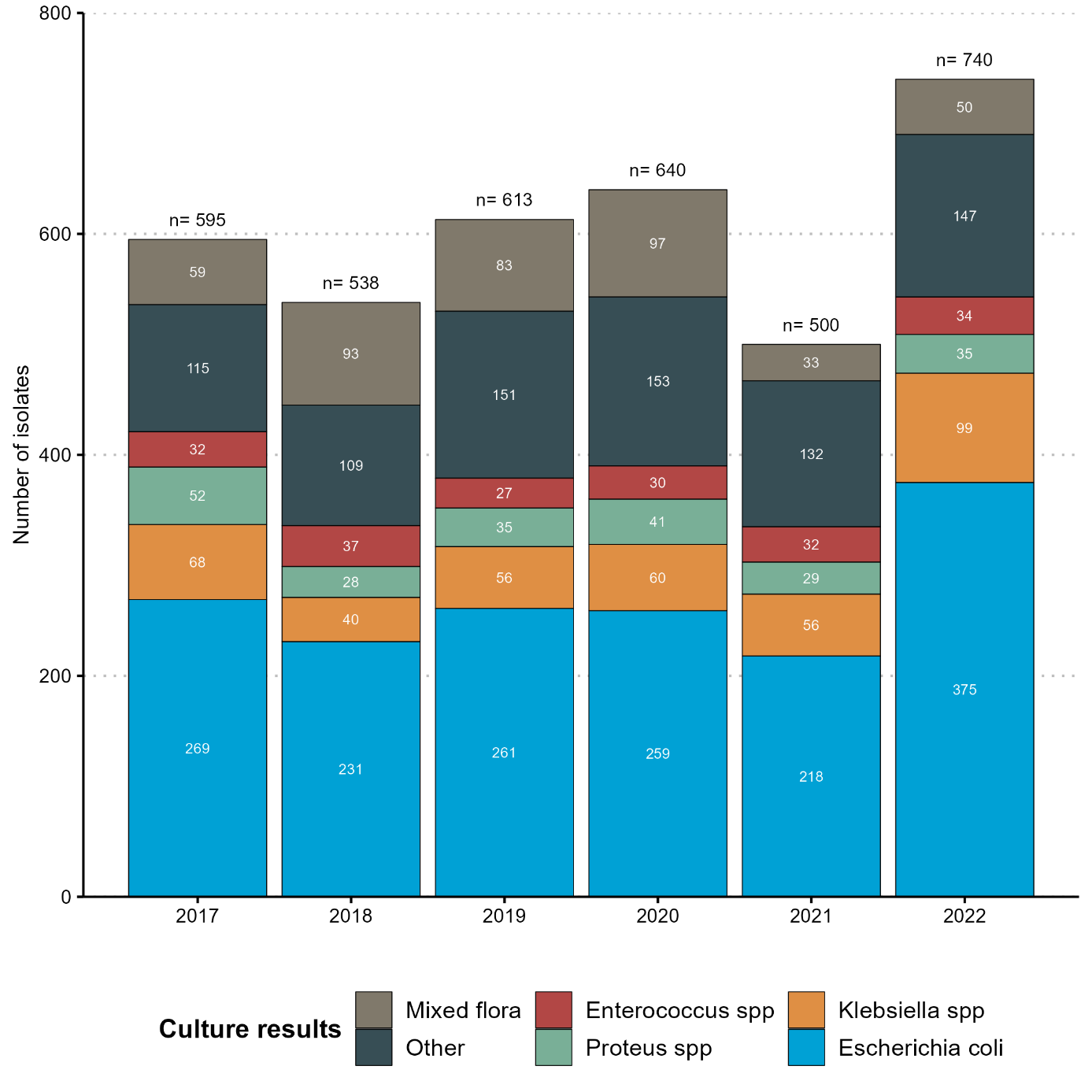


Figure S2: Overall 3’626 isolates from 3’056 cultures.

Mixed flora (n=415): cultures positive for > 3 microorganisms. Other microorganisms (n=807) included: *Streptococcus* spp. (n=207), other Gram positive cocci (n=134), *Pseudomonas* spp. (n=108), other Gram positive bacteria (n=96), *Staphylococcus* spp. (n=92), *Enterobacter* spp.(n=71), other enterobacteria (n=67), other Gram negative non-fermenters (n=13), *Candida* spp. (n=10), *Serratia* spp. (n=4), anaerobic bacteria (n=4), non-identifiable (n=1). spp.: Species

**Figure S3.** Frequency of bacteria of interest and corresponding counts and percentages of ESC-R expressing bacteria in the 43 NHs with incomplete data


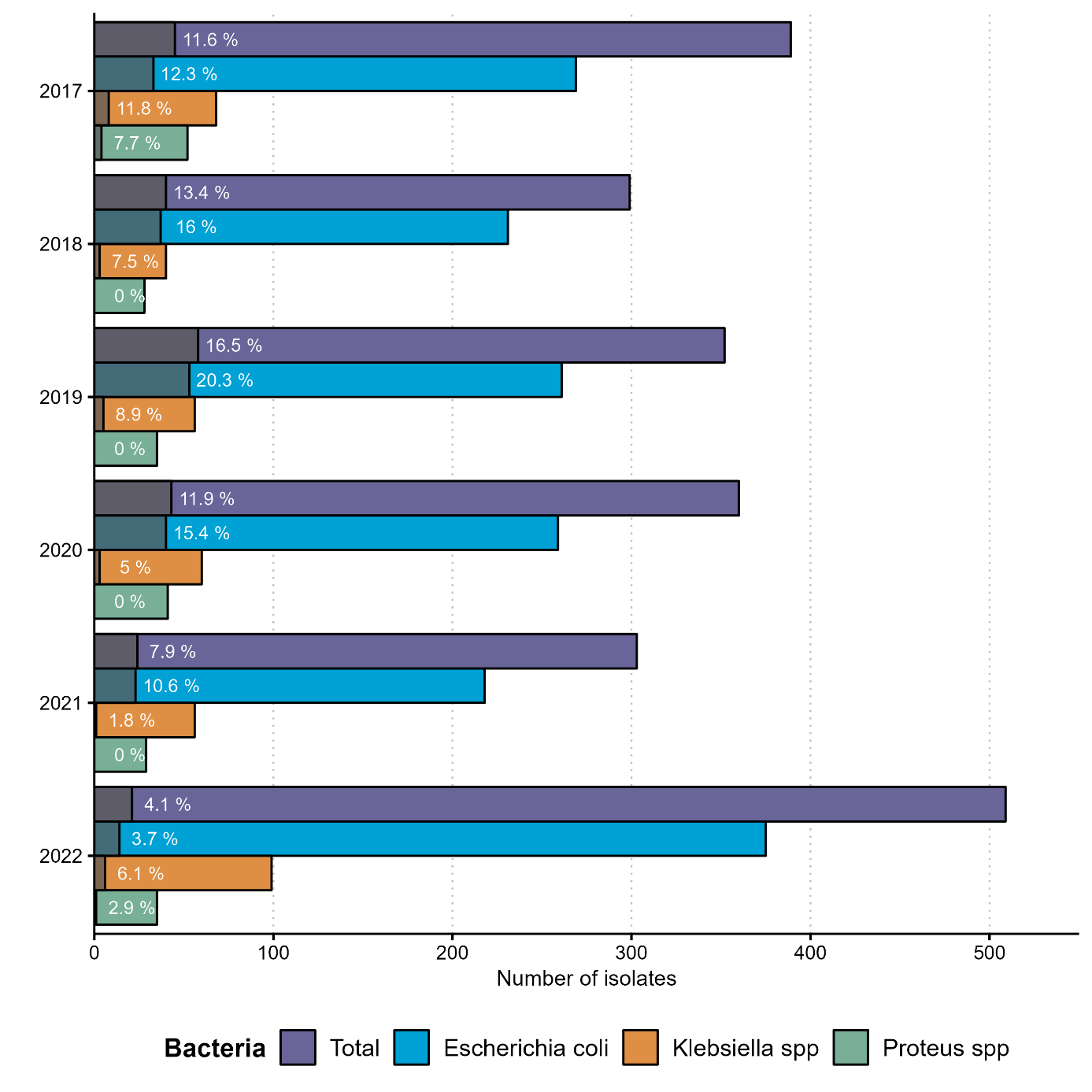


Figure S3: Shaded colors inside bars refer to counts and percentage labels to percentages of corresponding ESC-R bacteria. ESC-R: Extended-spectrum cephalosporin resistance, spp.: Species

**Figure S4.** Crude cumulative antibiotic consumption in the 54 NHs with complete data over the 6-year period


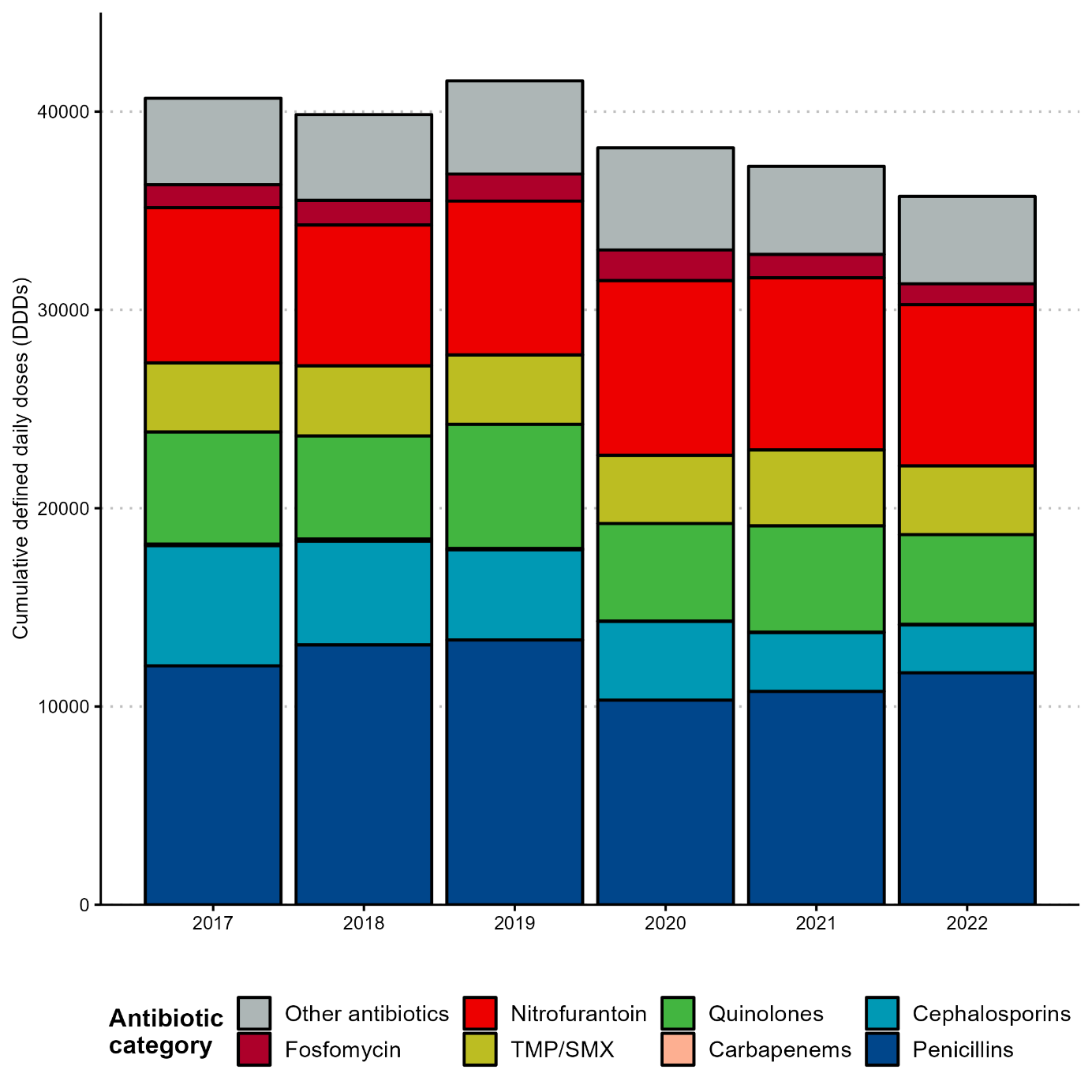


Figure S4: NH: Nursing home, TMP/SMX: trimethoprim/sulfamethoxazole

**Figure S5.** Crude cumulative antibiotic consumption in the 43 NHs with incomplete data over the six-year period


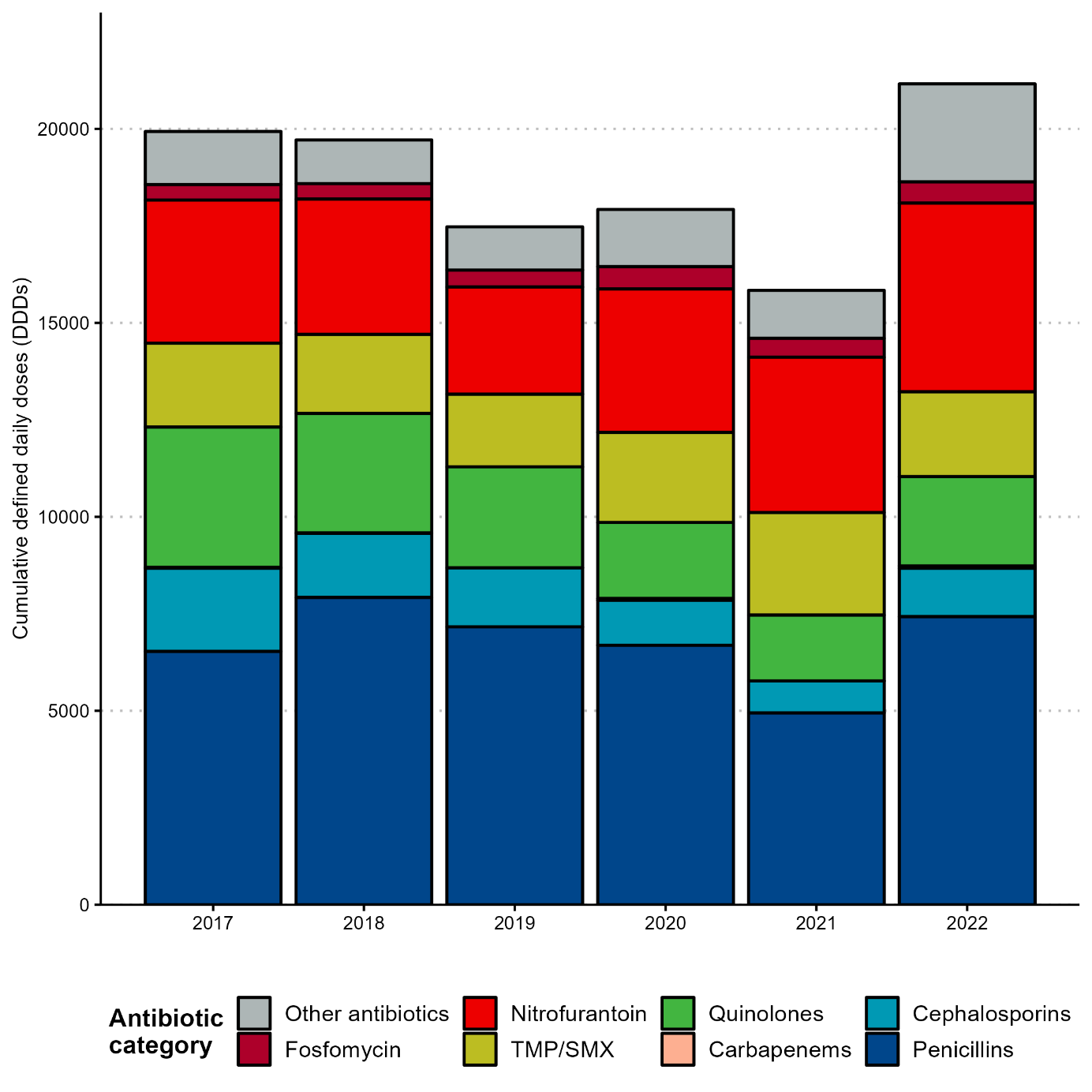


Figure S5: NH: Nursing home, TMP/SMX: trimethoprim/sulfamethoxazole

**Figure S6.** Relative consumption frequency of different antibiotic categories in the 43 NHs with incomplete data over the six-year period


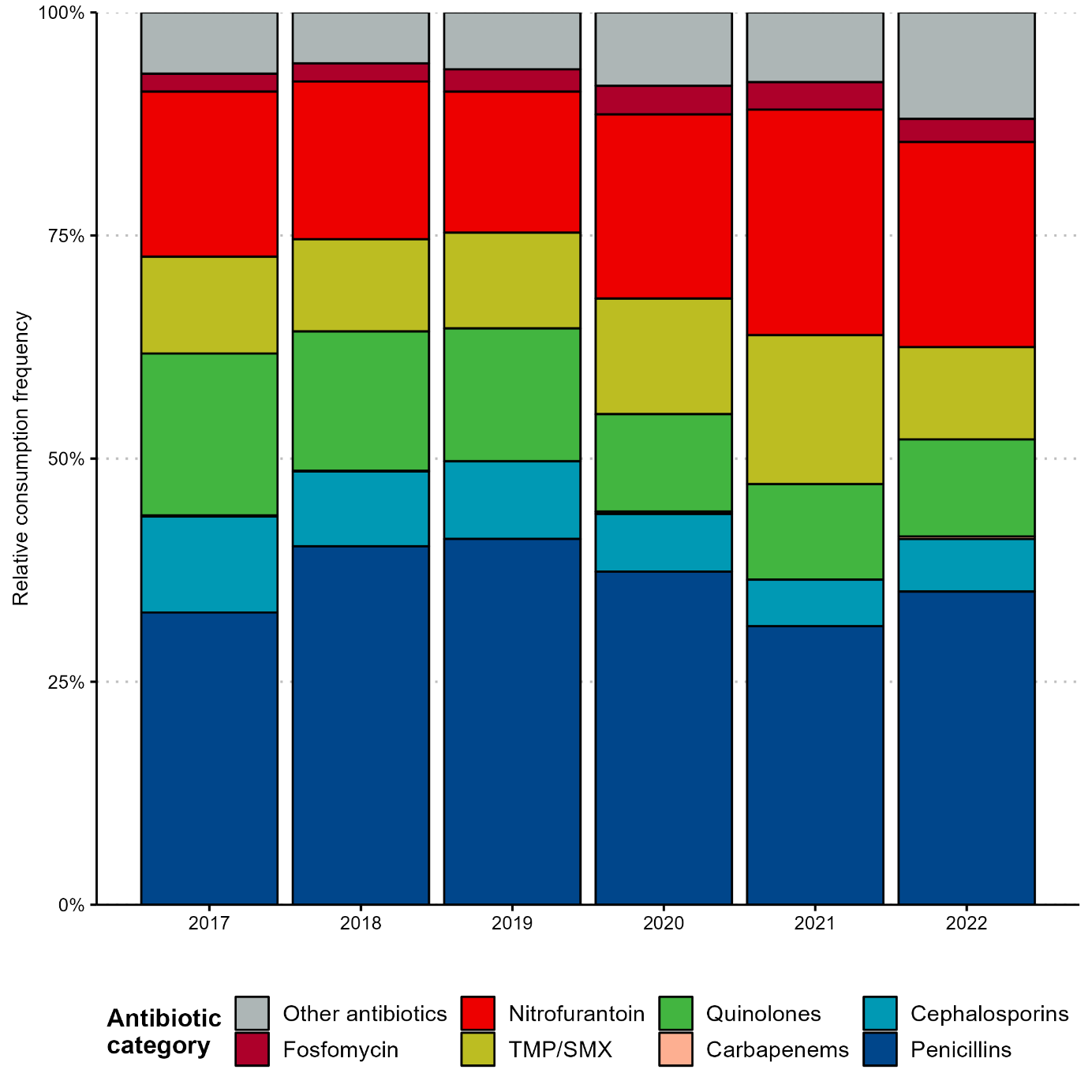


Figure S6: NH: Nursing home, TMP/SMX: trimethoprim/sulfamethoxazole

**SUPPLEMENTARY TABLES**

**Table S1.** Characteristics of nursing homes with complete, incomplete data, and overall

| **Year** | **All**  **NHs**  **(n= 97)** | **Complete data NHs**  **(n = 54)** | **Incomplete data NHs**  **(n = 43)** | ***p*-value^1^** |
| --- | --- | --- | --- | --- |
| ***Facility type, N (%)*** | | | | |
| **Geriatric** | 52 (53) | 26 (48) | 26 (60) | **0.04** |
| **Psychogeriatric** | 17 (18) | 7 (13) | 10 (23) |  |
| **Mixed** | 28 (29) | 21 (39) | 7 (17) |  |
| ***Localisation, N (%)*** | | | | |
| **Urban** | 59 (60) | 30 (56) | 29 (67) | 0.11 |
| **Rural** | 10 (11) | 4 (7) | 6 (14) |  |
| **Intermediate** | 28 (29) | 20 (37) | 8 (19) |  |

Table S1: 1: Chi-square test for comparisons between NHs with complete and incomplete data, n: Number, NH: Nursing home

**Table S2**. Annual number of residents and resident-days per NH among institutions with complete, incomplete data, and overall

| **Year** | **Number of residents**  **Median (IQR)**  **All**  **NHs**  **(n= 97^1^)** | **Number of residents**  **Median (IQR)**  **Complete data NHs**  **(n = 54^2^)** | **Number**  **of residents**  **Median (IQR)**  **Incomplete data NHs**  **(n = 43^3^)** | ***p*-value^4^** |  | **Resident- days Median (IQR)**  **All**  **NHs**  **(n= 97^1^)** | **Resident-days Median (IQR)**  **Complete data NHs**  **(n = 54^2^)** | **Resident-days**  **Median (IQR)**  **Incomplete data NHs**  **(n = 43^3^)** | ***p*-value^5^** |
| --- | --- | --- | --- | --- | --- | --- | --- | --- | --- |
| **2017** | 50.50  (39.80) | 57.00  (36.50) | 36.00  (29.20) | **<0.001** |  | 15’290  (11’919) | 16’961  (13’691) | 11’620  (9’270) | **<0.001** |
| **2018** | 49.50  (38.80) | 57.50  (36.00) | 37.50  (29.50) | **<0.001** |  | 15’376  (12’455) | 17’916  (12’530) | 10’820  (9’551) | **<0.001** |
| **2019** | 49.50  (37.20) | 57.50  (43.00) | 36.00  (22.00) | **<0.001** |  | 15’324  (11’847) | 17’530  (14’116) | 11’577  (6’995) | **<0.001** |
| **2020** | 49.50  (39.00) | 57.50  (49.50) | 37.50  (25.50) | **<0.001** |  | 14’834  (11’525) | 16’934  (15’248) | 11’506  (7’968) | **<0.001** |
| **2021** | 46.50  (38.00) | 55.50  (44.20) | 34.00  (21.50) | **<0.001** |  | 14’350  (11’921) | 17’775  (13’339) | 10’537  (7’453) | **<0.001** |
| **2022** | 48.00  (39.50) | 58.50  (38.80) | 39.00  (24.50) | **<0.001** |  | 14’876  (12’304) | 17’920  (11’008) | 11’613  (7’762) | **0.001** |

Table S2: 1: 91 units of analysis, 2: 50 units of analysis, 3: 41 units of analysis, 4: Wilcoxon test for comparisons of numbers of residents between NHs with complete and incomplete data, 5: Wilcoxon test for comparisons of numbers of resident-days between NHs with complete and incomplete data, IQR: Interquartile range, n: Number, NH: Nursing home

| **Year** | **Positive urinary cultures per resident**  **Median (IQR)**  **All**  **NHs**  **(n= 97^1^)** | **Positive urinary cultures per resident**  **Median (IQR)**  **Complete data NHs**  **(n = 54^2^)** | **Positive urinary cultures per resident**  **Median (IQR)**  **Incomplete data NHs**  **(n = 43^3^)** | ***p*-value^4^** |
| --- | --- | --- | --- | --- |
| **2017** | 0.274  (0.505) | 0.311  (0.602) | 0.125  (0.329) | 0.063 |
| **2018** | 0.357  (0.445) | 0.397  (0.485) | 0.250  (0.287) | 0.057 |
| **2019** | 0.374  (0.385) | 0.371  (0.368) | 0.396  (0.464) | 0.536 |
| **2020** | 0.462  (0.611) | 0.500  (0.515) | 0.190  (0.804) | 0.150 |
| **2021** | 0.255  (0.450) | 0.294  (0.382) | 0.154  (0.497) | 0.183 |
| **2022** | 0.426  (0.425) | 0.434  (0.283) | 0.348  (0.570) | 0.634 |

**Table S3.** Positive urinary cultures per resident per NH among institutions with complete, incomplete data, and overall

Table S3: 1: 91 units of analysis, 2: 50 units of analysis, 3: 41 units of analysis, 4: Wilcoxon test for comparisons between NHs with complete and incomplete data, IQR: Interquartile range, n: Number, NH: Nursing home

**Table S4.** Distributions of the percentage of ESC-R carrying bacteria among nursing homes with complete, incomplete data, and overall

| **Year** | **Percentage of ESC-R bacteria**  **Median (IQR)**  **All NHs**  **(n= 97^1^)** | **Percentage of ESC-R bacteria**  **Median (IQR)**  **Complete data NHs (n = 54^2^)** | **Percentage of**  **ESC-R bacteria**  **Median (IQR)**  **Incomplete data NHs (n = 43^3^)** | ***p*-value^4^** |
| --- | --- | --- | --- | --- |
| ***Overall*** | | | | |
| **2017** | 7.32%  (15.80) | 8.71%  (14.50) | 0.00%  (22.50) | 0.211 |
| **2018** | 4.65%  (14.00) | 5.44%  (12.80) | 0.00%  (17.50) | 0.752 |
| **2019** | 0.00%  (11.10) | 0.00%  (10.30) | 6.82%  (13.60) | 0.324 |
| **2020** | 3.12%  (10.60) | 2.86%  (10.80) | 3.12%  (9.52) | 0.891 |
| **2021** | 0.00%  (9.64) | 4.23%  (9.88) | 0.00%  (5.80) | 0.068 |
| **2022** | 1.41%  (8.82) | 3.34%  (9.58) | 0.00%  (4.57) | 0.084 |
| ***Escherichia coli*** | | | | |
| **2017** | 8.33%  (19.30) | 9.76%  (18.80) | 0.00%  (30.80) | 0.178 |
| **2018** | 6.26%  (17.50) | 6.90%  (16.70) | 0.00%  (23.80) | 0.675 |
| **2019** | 0.00%  (12.50) | 0.00%  (12.30) | 2.94%  (14.40) | 0.399 |
| **2020** | 2.78%  (13.10) | 1.43%  (13.10) | 3.53%  (13.30) | 0.775 |
| **2021** | 0.00%  (16.70) | 4.00%  (16.70) | 0.00%  (9.55) | 0.127 |
| **2022** | 0.00%  (10.50) | 4.55%  (13.80) | 0.00%  (3.60) | **0.014** |
| ***Klebsiella* spp.** | | | | |
| **2017** | 0.00%  (3.75) | 0.00%  (0.00) | 0.00%  (11.10) | 0.710 |
| **2018** | 0.00%  (0.00) | 0.00%  (0.00) | 0.00%  (0.00) | 0.827 |
| **2019** | 0.00%  (0.00) | 0.00%  (0.00) | 0.00%  (6.25) | **0.043** |
| **2020** | 0.00%  (0.00) | 0.00%  (0.00) | 0.00%  (5.00) | 0.221 |
| **2021** | 0.00%  (0.00) | 0.00%  (0.00) | 0.00%  (0.00) | 0.742 |
| **2022** | 0.00%  (0.00) | 0.00%  (0.00) | 0.00%  (0.00) | 0.167 |
| ***Proteus* spp.** | | | | |
| **2017** | 0.00%  (0.00) | 0.00%  (0.00) | 0.00%  (0.00) | 0.257 |
| **2018** | 0.00%  (0.00) | 0.00%  (0.00) | 0.00%  (0.00) | 0.574 |
| **2019** | 0.00%  (0.00) | 0.00%  (0.00) | 0.00%  (0.00) | 0.580 |
| **2020** | 0.00%  (0.00) | 0.00%  (0.00) | 0.00%  (0.00) | 0.640 |
| **2021** | 0.00%  (0.00) | 0.00%  (0.00) | 0.00%  (0.00) | 0.397 |
| **2022** | 0.00%  (0.00) | 0.00%  (0.00) | 0.00%  (0.00) | 0.207 |

Table S4: 1: 91 units of analysis, 2: 50 units of analysis, 3: 41 units of analysis, 4: Wilcoxon test for comparisons between NHs with complete and incomplete data, ESC-R: Extended-spectrum cephalosporin resistance, IQR: Interquartile range, n: Number, NH: Nursing home, spp.: Species

**Table S5.** Distributions of antibiotic consumption intensity among nursing homes with complete, incomplete data, and overall

| **Year** | **N of DDDs per 1000 resident-days**  **Median (IQR)**  **All NHs**  **(n= 97^1^)** | **N of DDDs per**  **1000 resident-days**  **Median (IQR)**  **Complete data NHs (n = 54^2^)** | **N of DDDs per 1000 resident-days**  **Median (IQR)**  **Incomplete data NHs (n = 43^3^)** | ***p*-value^4^** |
| --- | --- | --- | --- | --- |
| ***All antibiotics*** | | | | |
| **2017** | 39.30  (32.40) | 39.60  (29.30) | 39.00  (30.30) | 0.864 |
| **2018** | 36.40  (25.30) | 40.10  (17.80) | 30.00  (30.20) | 0.097 |
| **2019** | 38.80  (24.50) | 44.20  (21.00) | 32.60  (24.70) | 0.058 |
| **2020** | 33.80  (21.50) | 38.70  (19.20) | 29.00  (22.00) | 0.124 |
| **2021** | 37.60  (24.60) | 38.00  (18.10) | 34.90  (29.40) | 0.802 |
| **2022** | 35.00  (29.30) | 31.30  (26.70) | 36.80  (33.40) | 0.194 |
| ***Penicillins*** | | | | |
| **2017** | 11.90  (9.62) | 11.40  (9.10) | 12.60  (9.42) | 0.268 |
| **2018** | 13.80  (7.43) | 13.70  (7.41) | 14.40  (6.86) | 0.971 |
| **2019** | 12.00  (6.72) | 13.00  (6.12) | 11.60  (7.55) | 0.243 |
| **2020** | 9.99  (7.80) | 10.70  (6.95) | 8.91  (10.30) | 0.596 |
| **2021** | 9.56  (7.06) | 9.43  (6.59) | 9.78  (7.19) | 0.866 |
| **2022** | 10.90  (9.05) | 10.80  (6.74) | 12.80  (11.20) | 0.333 |
| ***Cephalosporins*** | | | | |
| **2017** | 3.67  (5.77) | 2.82  (6.11) | 3.70  (5.42) | 0.457 |
| **2018** | 2.26  (5.12) | 2.42  (7.33) | 2.03  (3.37) | 0.312 |
| **2019** | 2.65  (5.32) | 4.12  (5.06) | 2.32  (5.96) | 0.369 |
| **2020** | 1.90  (3.28) | 1.97  (2.82) | 1.84  (3.66) | 0.536 |
| **2021** | 1.51  (3.23) | 1.69  (2.83) | 1.14  (3.35) | 0.281 |
| **2022** | 1.66  (2.64) | 1.93  (2.46) | 1.26  (3.04) | 0.565 |
| ***Carbapenems*** | | | | |
| **2017** | 0.00  (0.00) | 0.00  (0.00) | 0.00  (0.00) | 0.226 |
| **2018** | 0.00  (0.00) | 0.00  (0.00) | 0.00  (0.00) | 0.567 |
| **2019** | 0.00  (0.00) | 0.00  (0.00) | 0.00  (0.00) | 0.086 |
| **2020** | 0.00  (0.00) | 0.00  (0.00) | 0.00  (0.00) | 0.723 |
| **2021** | 0.00  (0.00) | 0.00  (0.00) | 0.00  (0.00) | 0.345 |
| **2022** | 0.00  (0.00) | 0.00  (0.00) | 0.00  (0.00) | 0.920 |
| ***Quinolones*** | | | | |
| **2017** | 4.86  (5.59) | 4.31  (4.73) | 5.98  (7.22) | 0.459 |
| **2018** | 4.27  (4.14) | 4.68  (3.89) | 3.64  (4.41) | 0.472 |
| **2019** | 4.75  (5.81) | 4.70  (5.33) | 4.77  (6.09) | 0.403 |
| **2020** | 4.13  (4.78) | 4.59  (3.61) | 3.39  (4.65) | 0.182 |
| **2021** | 4.45  (4.28) | 4.57  (4.73) | 3.74  (3.80) | 0.332 |
| **2022** | 3.62  (4.28) | 3.64  (4.04) | 2.94  (4.84) | 0.941 |
| ***Trimethoprim-sulfamethoxazole*** | | | | |
| **2017** | 1.55  (3.91) | 2.49  (4.70) | 1.32  (3.17) | 0.264 |
| **2018** | 1.72  (4.11) | 2.06  (4.10) | 1.52  (3.83) | 0.323 |
| **2019** | 2.86  (4.35) | 2.91  (3.94) | 2.80  (4.54) | 0.620 |
| **2020** | 2.37  (4.60) | 2.70  (5.02) | 1.93  (3.97) | 0.104 |
| **2021** | 2.90  (5.06) | 3.01  (4.85) | 2.40  (5.53) | 0.877 |
| **2022** | 2.21  (4.58) | 2.18  (3.93) | 2.93  (5.06) | 0.461 |
| ***Nitrofurantoin*** | | | | |
| **2017** | 5.49  (7.89) | 4.79  (7.71) | 5.87  (10.70) | 0.920 |
| **2018** | 4.80  (8.66) | 5.43  (8.47) | 4.00  (8.08) | 0.178 |
| **2019** | 5.19  (8.83) | 6.25  (8.46) | 3.23  (7.81) | 0.092 |
| **2020** | 7.76  (9.34) | 7.76  (8.70) | 7.53  (9.23) | 0.393 |
| **2021** | 8.17  (10.70) | 7.85  (9.74) | 8.73  (13.60) | 0.927 |
| **2022** | 7.23  (10.90) | 7.06  (10.80) | 7.61  (10.80) | 0.681 |
| ***Fosfomycin*** | | | | |
| **2017** | 0.72  (0.98) | 0.74  (0.83) | 0.63  (1.12) | 0.349 |
| **2018** | 0.65  (1.01) | 0.71  (1.26) | 0.58  (0.96) | 0.419 |
| **2019** | 0.73  (1.23) | 0.81  (1.52) | 0.55  (1.00) | 0.232 |
| **2020** | 0.83  (1.42) | 1.00  (1.45) | 0.67  (1.38) | 0.176 |
| **2021** | 0.63  (1.45) | 0.60  (0.93) | 0.75  (1.86) | 0.902 |
| **2022** | 0.68  (1.17) | 0.74  (0.99) | 0.61  (1.35) | 0.990 |

Table S5: 1: 91 units of analysis, 2: 50 units of analysis, 3: 41 units of analysis, 4: Wilcoxon test for comparisons between NHs with complete and incomplete data, DDD: Defined daily dose, IQR: Interquartile range, n: Number, NH: Nursing home
